# The susceptibility to fosfomycin of Gram-negative bacteria isolates from urinary tract infection in the Czech Republic: data from a unicentric study

**DOI:** 10.1186/s12894-017-0222-6

**Published:** 2017-04-26

**Authors:** Miroslav Fajfr, Miroslav Louda, Pavla Paterová, Lenka Ryšková, Jaroslav Pacovský, Josef Košina, Helena Žemličková, Miloš Broďák

**Affiliations:** 1 Institute of Clinical Microbiology, University Hospital Hradec Kralové, Sokolska 581, Hradec Králové, 50005 Czech Republic; 2 Charles University in Prague, Faculty of Medicine in Hradec Kralové, Simkova 870, Hradec Kralove, 500 38 Czech Republic; 3 Clinic of Urology, University Hospital Hradec Kralové, Sokolska 581, Hradec Králové, 50005 Czech Republic

**Keywords:** Fosfomycin, Urinary tract infection, Susceptibility

## Abstract

**Background:**

Against a background of rapid increase of β-lactamase-producing or multi-resistant pathogenic bacteria and the resulting lack of effective antibiotic treatment, some older antibiotics have been tested for new therapeutic uses. One of these is fosfomycin, to which according to studies these resistant bacteria are very sensitive. Our study was designed because there is no data on the fosfomycin susceptibility rate in the Czech Republic.

**Method:**

In this study from January 2013 to June 2014 3295 unique isolates of Gram-negative bacteria which had caused urinary tract infections were examined. The antibiotic susceptibility was measured by disk diffusion test. Both EUCAST and CLSI guidelines criteria (for fosfomycin only) were used for the antibiotic susceptibility evaluation.

**Results:**

The most frequently tested bacterial isolates were *Escherichia coli* (51.3%, *n* = 1703), *Klebsiella pneumoniae* (19.4%, *n* = 643) and *Proteus* spp. (11.8%, *n* = 392). Among all isolates 29.0% (*n* = 963) were resistant to fluoroquinolones, 11.3% (*n* = 374) produced extended spectrum β-lactamase and 4.2% (*n* = 141) produced AmpC β-lactamase. The overall in vitro susceptibility was significantly higher for fosfomycin compared to the other tested per-oral antibiotics (nitrofurantoin, ampicillin, co-trimoxazole, ciprofloxacin and cefuroxime) against all tested Gram-negative rod isolates (excluding *Morganella morgani* and *Acinetobacter* spp. isolates). Fosfomycin also remained highly active against those isolates with extended spectrum β-lactamase (ESBL) production (95.8% in *Escherichia coli* isolates and 85.3% in *Klebsiella pneumoniae* isolates), unlike other tested per-oral antibiotics, which showed significant (*p* < 0.0001) susceptibility decrease.

**Conclusion:**

We have confirmed in the Czech Republic the very high susceptibility to fosfomycin trometamol of urinary tract infection pathogens, particularly Gram-negative rods including those producing β-lactamase.

## Background

Fosfomycin (Phosphomycin) as a new antimicrobial substance was first introduced in 1969. It is characterized as an anti-cell wall bactericidal antibiotic with a wide spectrum of antimicrobial activity, both to Gram-negative and Gram-positive bacteria [1]. It was used for many years as a highly effective antimicrobial drug especially for the treatment of urinary tract infections (UTIs), but with the advent of new antibiotics such as β-lactams or fluoroquinolones, it became somewhat obsolete. In the past decade there have been reports of a rapid increase in resistant pathogens, including extended spectrum β-lactamase (ESBL) producers or multi-drug resistant (MDR) pathogens (defined as non-susceptible to at least one agent in three or more antimicrobial categories) [2, 3]. Due to the lack of an effective antimicrobial drug for these cases, some older antibiotics were tested to evaluate their effectivity against multi-resistant bacteria. One such was fosfomycin, which according to the results of previously published studies had shown very good in vitro activity against resistant bacteria such as ESBL-producers, carbapenem-resistant *Klebsiella pneumoniae*, multi-resistant *Pseudomonas aeruginosa*, vancomycin-resistant enterococci (VRE) and methicillin-resistant *Staphylococcus aureus* (MRSA) [4–6]. All this evidence has generated higher interest in the use of fosfomycin in the last 5 years.

Fosfomycin for a long time has not been available in the Czech Republic, but since October 2014 has been available as the per-oral formulation, fosfomycin trometamol. Since there was no data on the susceptibility to fosfomycin of Czech bacterial isolates, we carried out this one-and-half year study with the aim of determining the fosfomycin susceptibility of isolates collected from UTIs among hospitalised and ambulatory patients in the University Hospital, Hradec Kralove.

## Methods

The sampling selection criteria for inclusion in the study allowed only samples from the urinary tract (urine, urethral swabs and samples from nephrostomies) examined in the Department of Clinical Microbiology, University Hospital Hradec Kralove. All Gram-negative bacterial isolates of significant quantity according to the European Association of Urology Guidelines 2015 [7] were collected throughout the whole study period (from January 2013 to June 2014). All duplicate isolates (the same bacterial isolates in significant quantity with the same antibiotic susceptibility in the same patient) were excluded. In total 3295 unique bacterial isolates were included. The isolates were from both hospitalised patients (55.1%, *n* = 1814) and hospital ambulant patients with a previous history of hospitalization (especially patients with chronic renal failure and patients after kidney transplantation) (44.9%, *n* = 1481). Our patients group did not include patients from the community. All study participants provided informed consent. The samples included in our research were processed strictly anonymously and therefore the approval of our ethical committee was not required. Nevertheless all sample processing and data evaluation were in compliance with the Helsinki Declaration.

### Bacterial culture and identification

Bacteria were cultured in 5% sheep blood agar and MacConkey agar and then tested for antibiotic susceptibility. Bacterial identifications were made by short biochemical line test (TRIOS®) or by a Biotyper Brucker® Matrix-Assisted Laser Desorption Ionization Time-of-Flight Mass Spectrometry (MALDI-TOF-MS) device according to standard operational procedures.

### Susceptibility testing

The susceptibility to fosfomycin, nitrofurantoin (in *E. coli* and *K. pneumoniae* isolates only), ampicillin, ampicillin/sulbactam (as a representative of amino-penicillins with β-lactamase inhibitors), trimethoprim-sulfamethoxazole (co-trimoxazole), ciprofloxacin and cefuroxime was determined by the disk diffusion method according to EUCAST guidelines. With the exception of fosfomycin, the EUCAST clinical breakpoints were used for interpretation of results [8]. For *K. pneumoniae* and nitrofurantoin susceptibility, the epidemiological cut-off value (ECOFF) 8 mm was used to distinguish susceptible and resistant isolates. All pathogens naturally resistant to tested antimicrobial substances were classified as resistant. As the current EUCAST version (2016) has no breakpoint available for the disk diffusion method for fosfomycin, the CLSI guidelines were used for interpretation of fosfomycin results [9]. The ESBL, AmpC or K1 β-lactamase producers were identified using the modified double disk synergy method according to the national recommendation [10]. For the ESBL quality control testing the *Klebsiella pneumoniae* strain ATCC700603 was used according to EUCAST guidelines. For AmpC quality control testing no testing strain is recommended in the EUCAST guidelines.

### Statistical methods

Chi-square (*χ* ^2^) test in software STATISTICA CZ 12 (StatSoft®, USA) was used for statistical analysis. *P*-value was used for comparison of antibiotic susceptibility and significance levels in all analyses were taken to be *p* ≤ 0.05. For the determination of the probability of inadequate antimicrobial coverage a weighted average was calculated of non-susceptibility for all uropathogens combined together in each of the patient groups [11].

## Results

### The bacterial isolates distribution

The most frequently found bacteria were *E. coli* (*n* = 1703, 51.3%), followed by *K. pneumoniae* (*n* = 643, 19.4%), *Proteus* species (*P. mirabilis* and *P. vulgaris*) (*n* = 392, 11.8%), and *Enterobacter* species (*E. cloacae*, *kobei*, *asburiae* and *aerogenes*) (*n* = 261, 7.9%). The other isolated Gram-negative bacteria were *Citrobacter* species (*n* = 97), *Morganella morganii* (*n* = 68), *P. aeruginosa* (*n* = 22) and *Providencia* species (*n* = 34). Of all the examined isolates 29.0% (*n* = 963) were resistant to ciprofloxacin. Overall 11.4% (*n* = 374) of isolates produced ESBL (mostly *K. pneumoniae*, *n* = 216), and 4.3% (*n* = 141) of isolates produced AmpC β-lactamase. In 414 cases (12.5% of all isolates) coproduction of AmpC or ESBL with resistance to ciprofloxacin was detected.

### Susceptibility testing results

*Escherichia* isolates showed very good susceptibility against fosfomycin (97.0%), nitrofurantoin (96.6%) and cefuroxime (90.5%), but poorer susceptibility against other common first line antibiotics – co-trimoxazole (67.8%) and ciprofloxacin (75.8%). *Klebsiella* strains showed good susceptibility only against fosfomycin (80.4%), and other tested first line antibiotics were poorly active (from 52.4% to 43.5%). Fosfomycin was also the most active antibiotic for *Enterobacter* isolates (82.8% against 77.2% - 0.0% susceptibility for the other tested first line antibiotics). Other Gram-negative bacterial isolates were also highly susceptible to fosfomycin with the exception of *Providencia* (susceptible 44.1%) and *Morganella* (susceptible only 16.2%). Other susceptibility results for the main groups of Gram-negative bacteria are presented in Table 1.

**Table 1:** Overall in-vitro susceptibility of the main Gram-negative rods to commonly used per-oral antibiotics (N/D - not defined susceptibility according to used EUCAST guidelines)

	Fosfomycin		Nitrofurantoin		Ampicillin		Ampicillin-sulbactam		Cefuroxime		Ciprofloxacin		Co-trimoxazole	
	*S%*	*R%*	*S%*	*R%*	*S%*	*R%*	*S%*	*R%*	*S%*	*R%*	*S%*	*R%*	*S%*	*R%*
*E. coli (n = 1703)*	97.0	2.2	96.6	3.4	46.3	53.7	76.3	23.7	90.5	9.5	75.8	24.0	67.8	31.8
*K. pneumoniae (n = 643)*	80.4	10.0	64.9	35.1	0.0	100.0	43.5	56.5	51.9	48.1	52.4	47.0	49.4	50.3
*Proteus sp.(n = 392)*	78.3	16.6	N/D	N/D	38.8	61.2	84.5	15.5	81.6	18.4	68.9	28.8	51.3	47.7
*Enterobacter sp.(n = 261)*	82.8	11.1	N/D	N/D	0.0	100.0	0.0	100.0	N/D	N/D	77.2	19.7	71.4	27.8
*Citrobacter sp. (n = 97)*	100.0	0.0	N/D	N/D	0.0	100.0	40.2	59.8	N/D	N/D	90.7	9.3	76.3	23.7
*M. morganii (n = 68)*	16.2	75.0	N/D	N/D	0.0	100.0	0.0	100.0	N/D	N/D	72.1	22.0	61.8	33.8
*Providencia sp. (n = 34)*	44.1	50.0	N/D	N/D	0.0	100.0	0.0	100.0	N/D	N/D	61.8	38.2	73.5	26.5

*E. coli Escherichia coli, K. pneumoniae Klebsiella pneumoniae, Proteus sp. Proteus species, Enterobacter sp. Enterobacter species, Citrobacter sp. Citrobacter species, M. morganii Morganella morganii, Providencia sp. Providencia species*

### Antibiotic susceptibility in common susceptible isolates and isolates producing β-lactamase (ESBL or AmpC)

For *E. coli* isolates only two antibiotics remained highly active against both common susceptible and β-lactamase producing isolates (ESBL or AmpC) – fosfomycin (respectively 97.4% and 92.0%) and nitrofurantoin (respectively 97.1% and 89.6%). The susceptibility of β-lactamase producing isolates was significantly lower (*p* < 0.0001) for all other tested first line antibiotics. A very similar situation was found also for *Enterobacter* isolates, for which there is susceptibility of both groups only for fosfomycin (no statistically significant decrease in β-lactamase producing isolates, *p* = 0.1081). Fosfomycin remained the only highly effective antibiotic against β-lactamase producing *K. pneumoniae* isolates; for all other tested antibiotics, β-lactamase producing isolates showed statistically significantly lower susceptibility (*p* < 0.0001). Further comparisons of the commonly susceptible and β-lactamase producing isolates are presented in Table 2.

**Table 2:** - Comparison of the susceptibility to commonly used first-line antibiotics and chemotherapeutics of the common susceptible isolates with that of isolates producing β-lactamase (ESBL or AmpC)

Antibiotic	*Escherichia coli* (*n* = 1578)			*Escherichia coli* beta lactamase positive^a^ (*n* = 125)			*Klebsiella pneumoniae* (*n* = 359)			*Klebsiella pneumoniae* beta lactamase positive^a^ (*n* = 284)			*Enterobacter* species (*n* = 186)			*Enterobacter* species beta lactamase positive^a^ (*n* = 75)		
	S%	I%	R%	S%	I%	R%	S%	I%	R%	S%	I%	R%	S%	I%	R%	S%	I%	R%
Fosfomycin	97.4	0.8	1.8	92.0	1.6	6.4	85.8	5.6	8.6	73.6	14.8	11.6	86.1	4.8	9.1	74.7	9.3	16.0
Ampicilin	50.0	0.0	50.0	0.0	0.0	100.0	0.0	0.0	100.0	0.0	0.0	100.0	0.0	0.0	100.0	0.0	0.0	100.0
Ampicilin-sulbactam	82.4	0.0	17.6	0.0	0.0	100.0	78.3	0.0	21.7	0.0	0.0	100.0	0.0	0.0	100.0	0.0	0.0	100.0
Cefuroxime	97.7	0.0	2.3	0.0	0.0	100.0	92.8	0.0	7.2	0.0	0.0	100.0	0.0	0.0	100.0	0.0	0.0	100.0
Ciprofloxacin	80.6	0.3	19.1	16.0	0.0	84.0	84.5	1.2	14.3	12.0	0.0	88.0	88.7	1.1	10.2	49.3	8.0	42.7
Co-trimoxazole	71.0	0.4	28.6	28.0	0.0	72.0	80.5	0.3	19.2	10.2	0.4	89.4	84.9	0.0	15.1	38.6	2.7	58.7
Nitrofurantoin	97.1	0.0	2.9	89.6	0.0	10.4	76.6	0.0	23.4	50.0	0.0	50.0	65.1	0.0	34.9	65.3	0.0	34.7

^a^beta lactamase positive means bacterial isolates producing ESBL or AmpC beta lactamase

### Comparison of the susceptibility of the four most frequent Gram-negative bacterial isolates to fosfomycin with that of the other first line antibiotics

Fosfomycin showed relatively good activity against *Proteus* isolates which are primarily resistant to nitrofurantoin (82.5% of isolates were susceptible to fosfomycin). Additionally, *E. coli*, *K. pneumoniae* and *Enterobacter* species isolates resistant to nitrofurantoin remained susceptible to fosfomycin, between 78.6% and 88.1%. There was high resistance in all tested strains against ampicillin and ciprofloxacin; however, between 77.9% and 97.5% of ampicillin- or ciprofloxacin-resistant bacterial isolates were susceptible to fosfomycin.

### Comparison of overall susceptibility to first line antibiotics according to patient status

All samples were allocated to one of three groups – patients from intensive care units (ICUs), from standard hospital wards, and ambulatory. Resistance to all tested antibiotics was lowest in hospital ambulatory patients and highest in patients from ICUs. There was significantly higher resistance to all evaluated antibiotics in standard wards and ICUs compared with hospital out-patients (*p* = 0.006 to *p* < 0.0001), with one exception: there was lower resistance to fosfomycin in all groups (*p* = 0.1173 and *p* = 0.2334). Full data are presented in Table 3.

**Table 3:** The comparison of overall antibiotic resistance according to patient status - patients from intensive care units (ICU), standard hospital wards (SHW) or from hospital ambulance (AMB)

		AMP-R (%)	AMS-R (%)	CRX-R (%)	CIP-R (%)	COT-R (%)	FUR-R (%)	FOS-R (%)
ICU	*n* = 639	79.3	47.7	40.1	35.4	45.4	33.0	11.3
SHW	*n* = 1175	75.6	44.0	33.2	37.9	44.1	30.6	7.8
AMB	*n* = 1481	63.8	32.3	25.7	17.7	26.8	25.7	9.1
Statistical evaluation (*p*-value)								
Ambulance versus intensive care units		<0.0001	< 0.0001	< 0.0001	< 0.0001	< 0.0001	0.0060	0.1173
Ambulance versus standard hospital wards		< 0.0001	< 0.0001	< 0.0001	< 0.0001	< 0.0001	0.,0052	0.2334

*AMP-R* ampicillin resistance, *AMS-R* ampicillin-sulbactam resistance, *CRX-R* cefuroxime resistance, *CIP-R* ciprofloxacin resistance, *COT-R* co-trimoxazole resistance, *FUR-R* nitrofurantoin resistance, *FOS-R* fosfomycin resistance

## Discussion

The increasing incidence of urinary tract infection caused by Gram-negative bacteria with multiple drug resistance is well described worldwide and also by the European Antimicrobial Resistance Surveillance Network (EARS-Net), where this phenomenon has been reported in *E. coli* and *K. pneumoniae* isolates [12, 13]. The same data were obtained in our study: for example, more than 47% of *K. pneumoniae* isolates were resistant to fluoroquinolones, and 44% produce β-lactamase (ESBL or AmpC). These findings underlie the necessity for the increasing use of highly effective parenteral antibiotics such as aminoglycosides, 3^rd^ generation cephalosporins or carbapenems in urinary tract infection treatment, and which also often requires hospitalisation. Per-oral treatment is mainly confined to mild infections such as uncomplicated cystitis. Our study compared the susceptibility to commonly-used first line antibiotics (fosfomycin, nitrofurantoin, ampicillin, ampicillin-sulbactam, co-trimoxazole, ciprofloxacin, cefuroxime) of susceptible bacterial isolates and bacterial isolates evincing multiple resistance (ESBL or AmpC), with the aim of determining the feasibility of using per-oral antibiotics (especially fosfomycin trometamol) in the therapy of urinary tract infection caused by multiply-resistant pathogens.

Our study confirmed the leading role of *E. coli* in urinary tract infection, as nearly 51.0% of urinary samples contained this pathogen. The other most frequent isolates are also of Gram-negative rods, especially *Klebsiella*, *Proteus* or *Enterobacter*, which together comprised 40.0% of all tested isolates. The results were in congruence with studies from other countries [14, 15]. *E. coli* isolates were highly susceptible to fosfomycin and nitrofurantoin (97.0% and 96.6% respectively) and less susceptible to ciprofloxacin and trimethoprim-sulfamethoxazole (75.8% and 67.8%), which is in line with other studies from Europe and Asia [13, 15, 16]. A similar pattern was found in *K. pneumoniae,* where 80.4% of isolates were susceptible to fosfomycin, although other tested antibiotics showed susceptibility in the range 43.5% to 64.4%. In β-lactamase producing bacterial isolates (ESBL or AmpC), fosfomycin showed the lowest proportion of resistant isolates. However, it is necessary to comment that comparison of antibiotics and chemotherapeutics is sometimes difficult due to the absence of EUCAST break points for many of them. A good example is nitrofurantoin, which according to EUCAST guidelines has a susceptibility range only for *E. coli*, but which according to CLSI guidelines has assessed break points for all *Enterobacteriacae*. The problematic assessment of susceptibility testing according to different guidelines is well known [17]. The good activity of fosfomycin against extended spectrum β-lactamase positive Gram-negative bacteria in comparison with other first line antibiotics has also been observed in other countries [15, 16, 18–20].

The evaluation of overall antibiotic susceptibility in relation to patient status revealed significantly lower resistance in bacterial isolates from ambulatory patients in comparison to those of hospitalized patients. With the exception of ICU isolates, and using resistance of 10% as the limit for use as a first line option for empirical therapy in non-life-threatening infections, only fosfomycin seems to have an adequate coverage amongst agents suitable for per oral therapy. In our study there were no samples from the community (from GPs), which is why our samples had higher resistance.

Our results showed fosfomycin trometamol as a promising antibiotic for urinary tract infection in our country. But according to some published studies, the increasing use of fosfomycin worldwide has led to increased levels of resistance in isolates. For example, in a Spanish study an increase of fosfomycin resistance was detected in *Escherichia coli* isolates from 0.0% in 2005 to 14.4% in 2011 [21]. A change in the guidelines for UTI therapy is required to prevent this side effect of mass use of fosfomycin trometamol for UTIs. We see the main future benefit of a per-oral fosfomycin formulation in our fosfomycin-naive population in the treatment of specific groups of patients with non-life-threatening urinary tract infections, such as in patients with long-term stents which were often colonised by multi-resistant Gram-negative bacteria. These patients are threatened frequently by chronic urinary tract infections, and after successful intra-venous antibiotic therapy during hospitalization they should be treated by continuous per-oral therapy at home. Of all the commonly available per-oral antibiotics only fosfomycin was shown in our study to be highly effective against multi resistant (ESBL or AmpC) Gram-negative bacteria.

## Conclusion

We have confirmed in the Czech Republic the very high susceptibility to fosfomycin trometamol of urinary tract infection pathogens, particularly Gram-negative rods including those producing β-lactamase. Our results will be used to rationalise treatment of urinary tract infection by fosfomycin trometamol in our country.

## Abbreviations

CLSI: The clinical and laboratory standards institute
EARS-Net: The European Antimicrobial Resistance Surveillance Network
ECOFF: The epidemiological cut-off value
ESBL: Extended spectrum β-lactamase
EUCAST: The European Committee on Antimicrobial Susceptibility Testing
ICU: Intensive care unit
MALDI-TOF-MS: Matrix-assisted laser desorption ionization time-of-flight mass spectrometry
MDR: Multi-drug resistance
MRSA: Methicillin-resistant *Staphylococcus aureus*
UTI: Urinary tract infection
VRE: Vancomycin-resistant enterococci
